# Decolorization of a recalcitrant organic compound (Melanoidin) by a novel thermotolerant yeast, *Candida tropicalis* RG-9

**DOI:** 10.1186/1472-6750-12-30

**Published:** 2012-06-18

**Authors:** Soni Tiwari, Rajeeva Gaur, Ranjan Singh

**Affiliations:** 1 Department of Microbiology (Centre of Excellence), Dr. Ram Manohar Lohia Avadh University, Faizabad, 224001, Uttar Pradesh, India; 2 Amity Institute of Microbial Biotechnology, Amity University, Noida, G. B. Nagar, Uttar Pradesh, India

**Keywords:** Melanoidin, Decolorization, Thermotolerant, *Candida tropicalis*, Static condition

## Abstract

**Background:**

Sugarcane distilleries use molasses for ethanol production and generate large volume of effluent containing high biological oxygen demand (BOD) and chemical oxygen demand (COD) along with melanoidin pigment. Melanoidin is a recalcitrant compound that causes several toxic effects on living system, therefore, may be treated before disposal. The aim of this study was to isolate a potential thermotolerant melanoidin decolorizing yeast from natural resources, and optimized different physico-chemical and nutritional parameters.

**Results:**

Total 24 yeasts were isolated from the soil samples of near by distillery site, in which isolate Y-9 showed maximum decolorization and identified as *Candida tropicalis* by Microbial Type Culture Collection (MTCC) Chandigarh, India. The decolorization yield was expressed as the decrease in the absorbance at 475 nm against initial absorbance at the same wavelength. Uninoculated medium served as control. Yeast showed maximum decolorization (75%) at 45°C using 0.2%, glucose; 0.2%, peptone; 0.05%, MgSO4; 0.01%, KH_2_PO_4_; pH-5.5 within 24 h of incubation under static condition. Decolorizing ability of yeast was also confirmed by high performance liquid chromatography (HPLC) analysis.

**Conclusion:**

The yeast strain efficiently decolorized melanoidin pigment of distillery effluent at higher temperature than the other earlier reported strains of yeast, therefore, this strain could also be used at industrial level for melanoidin decolorization as it tolerated a wide range of temperature and pH with very small amount of carbon and nitrogen sources.

## Background

Existing population bang globally urges rise of industrial sectors resulting in pollution of water, air and soil. The release of pollutants into the environment from various industries causes hazard to living organisms resulting in a greater environmental stress. One such industry of fast development is the distillery industry. There are more than 295 distilleries in India, producing approximately 2.7 billion liters of alcohol and releasing 40 billion liters of spentwash (distillery effluent) annually [1].

Dark brown color of distillery spentwash is mainly due to the presence of organic compound known as melanoidin [2]. Melanoidin is main content of spentwash and is formed by the reaction between amino acid and carbohydrate called “Maillard reaction” [1,3]. These highly colored components hinder sunlight penetration in rivers, lakes or lagoons which inturn decrease both photosynthetic activity and dissolved oxygen concentration causing harm to aquatic life. Disposal of spentwash on land is equally hazardous causing a reduction in soil pH, inhibition of seed germination and potable water [4].

The colored compound in spentwash has antioxidant properties and become toxic to all living system including microorganisms, therefore, must be treated before disposal into environment [5,6]; melanoidin can be removed by several common physico-chemical methods. Still, these methods require high reagent dosages and generate large amount of sludge [7,8]. Biological methods present an incredible alternate for decolorization/degradation of spentwash due to their low cost, environmental friendly and publicly acceptable treatment and cost-competitive alternative to chemical decomposition processes [8,9].

A number of biological processes such as bioadsorption and biodegradation have been reported having prospective application in color removal from spentwash [10-16]. A wide variety of aerobic microorganisms capable of decolorizing spentwash include bacteria, fungi, cyanobacteria and yeasts. Some bacterial strains isolated from sewage and acclimatized on increasing concentrations of distillery waste, which were able to reduce chemical oxygen demand (COD) by 80% in 4–5 days without any aeration and the major products left after the degradation process were biomass, carbon dioxide and volatile acids [17]. Raghukumar and Rivonkar [18] isolated a marine fungus, *Flavodon flavus*, which was more effective in decolorizing raw molasses spentwash than was the molasses wastewater collected either after anaerobic treatment or after aerobic treatment. Tondee and Sirianutapiboon [19] isolated *Issatchenkia orientalis* yeast from fruit sample which showed 60% melanoidin decolorization at 30 °C in 7 days under aerobic condition.

In the present investigation, an attempt was made to isolate such strain from natural ecosystem which has ability to grow at higher temperature with minimum expense of simple sugar and higher percentage of melanoidin decolorization ability.

## Results and discussion

### Isolation, screening and identification of the isolates

A total of 24 yeast isolates capable of dye decolorization were isolated on the GPYE agar medium from the soil of distillery near by the Masudha distillery Faizabad, India. The isolates showing higher clear zone around the colony on GPYE agar were selected for further study (pH 5.5, 24–48 h and 45 °C). The clear zone diameter of more than 1 cm around the colony was considered as effective isolates for decolorization (data not shown).

For further study, isolates were inoculated in 50 ml of medium and incubated at 35°C and 45°C for 24–48 h for selection of thermotolerant melanoidin decolorizing yeast individually. Among yeast isolates, higher decolorization (67%) was shown by yeast isolate Y-9 identified by MTCC Chandigarh as *Candida tropicalis* RG-9. However, this isolate of yeast was separately optimized for higher decolorization at different medium with varying contents of carbon, nitrogen sources and their different concentrations.

**Figure 1:**
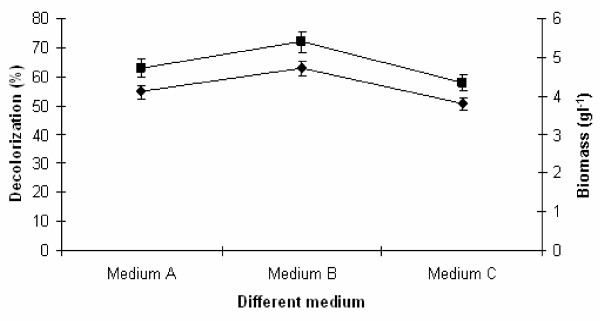
**Selection of efficient culture medium for melanoidin decolorization.** Decolorization yield (), Biomass production (). The inoculated flasks were contained three different medium at 37°C temperature for 24–48 h at static condition. Error bars presented are mean values of ± standard deviation of triplicates of three independent experiments.

The effect of medium composition on decolorization by yeast is clear as mentioned in Figure 1. Yeast strain showed higher melanoidin decolorization (67%) in medium B (0.5%, glucose; 0.2%, yeast extract; 0.3%, peptone; 0.05%, MgSO_4_; 0.05%, K_2_HPO_4_ with 3.5 OD effluent) when compared to medium A and C. Medium B was found most suitable because this medium could provide more organic form of nitrogen source than others. Therefore, nitrogen requirement by the isolate was higher for better decolorization, this could probably by improving metabolic activities for enzyme secretion or the biomass could be promoted. However, medium B was selected for optimization of physico-chemical and nutritional parameters for melanoidin decolorization by yeast strain Y-9 (Figure 1).

### Effect of different temperature on melanoidin decolorization

**Figure 2:**
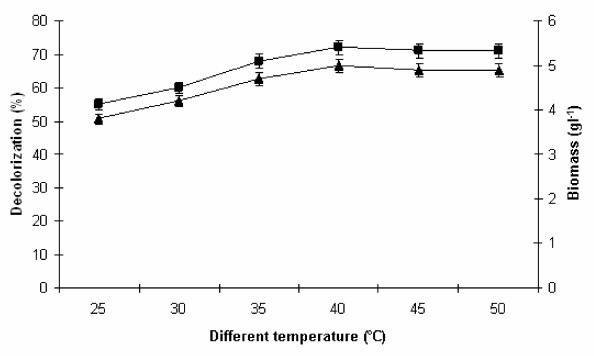
**Effect of different temperature on melanoidin decolorization.** Decolorization yield (), Biomass production (). The inoculated flasks were incubated at different temperature (°C) for 24–48 h at static condition in medium. Error bars presented are mean values of ± standard deviation of triplicates of three independent experiments.

The influence of temperature regime on melanoidin decolorization and biomass production was studied by varying the temperature from 25°C to 50°C while other parameters were maintained constant. From Figure 2 it was observed that melanoidin decolorization by yeast strain Y-9 was active at all temperatures employed with maximum decolorization at 40°C to 50°C. It exhibited 72% decolorization with 5.0 g l^−1^ biomass production. The remarkable decolorization (72%) in the temperature range of 40–50°C reveals thermotolerant as well as mesophilic nature of the yeast strain. Our strain showed better decolorization potential at higher temperature than Sirianuntapiboon et al. [20] who reported a maximum of 68% spentwash decolorization at 30°C by *Citeromyces* sp. WR-43-6. Similarly, Tondee and Sirianutapiboon. [19] reported that *Issatchenkia orientalis* showing maximum 60% spentwash decolorization at 30 °C. Further, increase in temperature could not affect the biomass production as well as decolorization efficiency by yeast strain Y-9. According to Cetin and Donmez [21], high temperature may cause loss in cell viability or deactivation of the enzymes responsible for decolorization resulted into suppressed decolorizing activity. Therefore, the melanoidin decolorization and biomass production efficiency of our strain Y-9 was certainly better than reported by other researchers. Thus, it may be suggested that the optimal temperature for melanoidin decolorization depends on the variation of microbial strains and their genetic diversity as they have been isolated from a very wide range of climatic conditions.

### Effect of different time course on melanoidin decolorization

**Figure 3:**
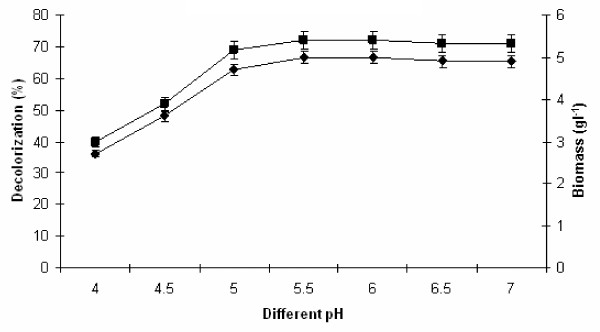
**Effect of different incubation periods on melanoidin decolorization.** Decolorization yield (), Biomass production (). The inoculated flasks were incubated at different incubation period at 45°C under static condition in medium. Error bars presented are mean values of ± standard deviation of triplicates of three independent experiments.

Time course of melanoidin decolorization was studied alongwith biomass production of yeast strain Y-9. Maximum decolorization (72%) was achieved in 24 h of incubation with 4.95 g l^−1^ biomass production (Figure 3). Further increase in the incubation period did not increase the decolorization. On contrary Tondee and Sirianutapiboon. [19] reported 60% decolorization by *Issatchenkia orientalis,* but after 7 days of incubation. In another study Sirianuntapiboon et al. [20] had been reported a maximum 68% decolorization using *Citeromyces* sp. WR-43-6 after 7 days of incubation. Therefore, decolorization and growth efficiency of our strain Y-9 is certainly better than that reported by other researchers.

### Effect of different pH on melanoidin decolorization

**Figure 4:**
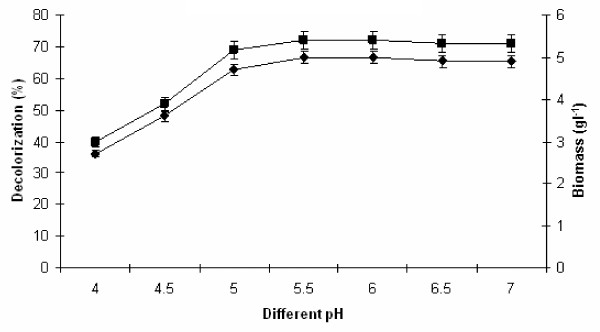
**Effect of different pH on melanoidin decolorization.** Decolorization yield (), Biomass production (). The inoculated flasks were incubated at different pH at 45°C for 24 h under static condition in medium. Error bars presented are mean values of ± standard deviation of triplicates of three independent experiments.

The influence of pH on melanoidin decolorization and biomass production was studied at varying pH from 4.0 to 7.0 at their optimal temperature and incubation period. Maximum 72% decolorization was achieved at pH 5.5 with 4.95 g l^−1^ biomass production (Figure 4). Strain Y-9 exhibited its optimal decolorization at pH 5.5 and any deviation from optimum level of pH reduced the melanoidin decolorization activity. The decrease in decolorization activity was drastic towards more acidic pH leading to no activity at pH 3.0–4.0. Melanoidin decolorization from other yeast strain was also reported by several researchers having maximum decolorization activity in 5.0–6.0 optimum pH range [19,20]. Several workers have studied that enzymes produced by microorganism during the decolorization were effective only in acidic conditions [22]. An increase in color at higher pH was due to the polymerization of melanoidin and higher rate nutrient utilization [23,24]. Similar results have been reported when soil samples were used as inoculum instead of isolated organisms [23,25,26]. Melanoidin decolorization got reduced at above and below of this pH due to inhibition of enzyme production as well as activity. All enzymes are protein in nature, therefore, some proteins denatured at higher or lower pH value. Each microorganism has a specific pH for their growth and enzyme activity in the surrounding environment. Therefore, the physiological function of yeast varies from strain to strain.

### Effect of different carbon sources on melanoidin decolorization

**Figure 5:**
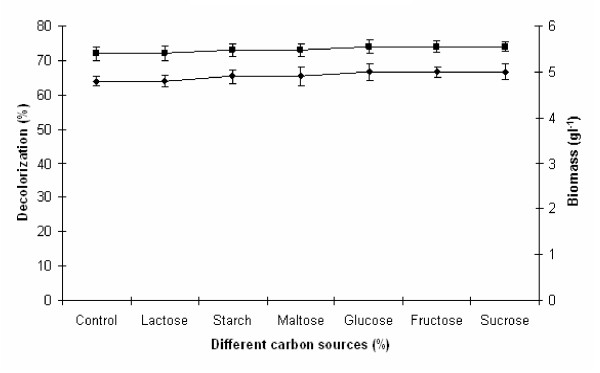
**Effect of different carbon sources on melanoidin decolorization.** Decolorization yield (), Biomass production (). The control flask does not contain any carbon sources. Test flasks contained different carbon sources in the medium at a level of 0.5% (w/v). Inoculated flasks were incubated at 45°C for 24 h. Error bars presented are mean values of ± standard deviation of triplicates of three independent experiments.

In another approach, the effect of various carbon sources (0.5%, w/v) on melanoidin decolorization and biomass production was also investigated for 24 h of incubation, and the results are depicted in Figure 5. Melanoidin decolorization by yeast strain Y-9 is extraordinarily stable in the presence of all carbon sources under study. It was observed that except lactose, presence of other carbon sources enhanced the melanoidin decolorization when compared to control (without carbon source). With control (without carbon source), sucrose, glucose, fructose and starch, yeast strain Y-9 showed 70, 72, 74, 73, and 72% decolorization, respectively. The presence of lactose marginally reduced decolorization. From Figure 5 it was observed that higher decolorization (74%) and biomass (5.0 g l^−1^) was reported by glucose when compared to control (without sugar), while fructose favored the decolorization. Melanoidin decolorization from other yeast are maximum in the presence of glucose have reported by others researchers also [19,20]. Watanabe et al. [27] have reported the enzymatic degradation of melanoidin by *Coriolus sp.* No. 20 having an intracellular enzyme, which required active oxygen molecules and sugars (sorbose as well as glucose) in reaction mixture, was later identified as sorbose oxidase which oxidize glucose into gluconic acid. It is, therefore, evident from our study that melanoidin decolorization by yeast strain Y-9 is remarkably stable in the presence of broad range of carbon sources employed in this study.

### Effect of different concentration of glucose on melanoidin decolorization

**Figure 6:**
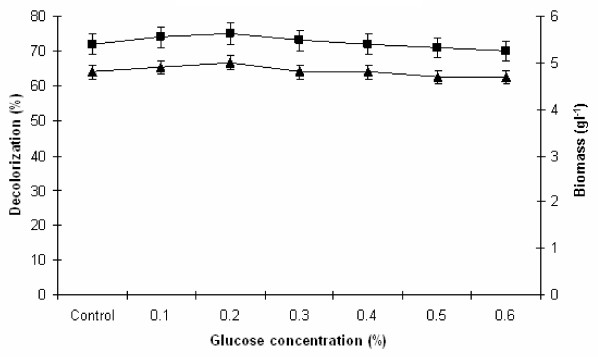
**Effect of different glucose concentration on melanoidin decolorization.** Decolorization yield (), Biomass production (). The control flask does not contain glucose. Test flasks contained different concentration of glucose in the medium at a level of 0.6% (w/v). Inoculated flasks were incubated at 45°C for 24 h. Error bars presented are mean values of ± standard deviation of triplicates of three independent experiments.

In another set of the experiment, the effect of various concentration of glucose (0.1, 0.2, 0.3, 0.4, 0.5 and 0.6%, w/v) on melanoidin decolorization and biomass production was also investigated and the results are depicted in Figure 6. Melanoidin decolorization by yeast strain Y-9 is extraordinarily stable in the presence of all glucose concentrations under study. It was observed that glucose concentration above 0.3% (w/v), decreased the melanoidin decolorization. From Figure 6 it was observed that maximum 75% decolorization with 5.0 g l^−1^ biomass production was achieved at 0.2% (w/v) glucose concentration, above this concentration decolorization reduced and biomass was slightly increased. This effect can be explained as during initial phase of growth organism utilizes easily available carbon sources added to the medium and then starts to degrade spentwash components for carbon source [11]. Tondee and Sirianutapiboon. [19] have also reported that *Issatchenkia orientalis* utilized 2.5% glucose for maximum decolorization (60%) and above this concentration of glucose there was decrease in the decolorization. Similarlly, Sirianuntapiboon et al. [20] have reported that *Citeromyces* sp. WR-43-6 showed 68% decolorization in the presence of 2.0% glucose concentration. Ohmomo et al. [10] have reported that glucose was the best carbon source, which utilized by *Aspergillus fumigatus* G-2-6 for maximum degradation of melanoidin and further increase in glucose concentration resulted in an increase in mycelial biomass but no further increase in decolorization yield. It is, therefore, evident from our study that melanoidin decolorization by yeast strain Y-9 is remarkably higher in the presence of 0.2% (w/v) glucose within 24 h of incubation when compared to other researchers.

### Effect of different nitrogen sources on melanoidin decolorization

**Figure 7:**
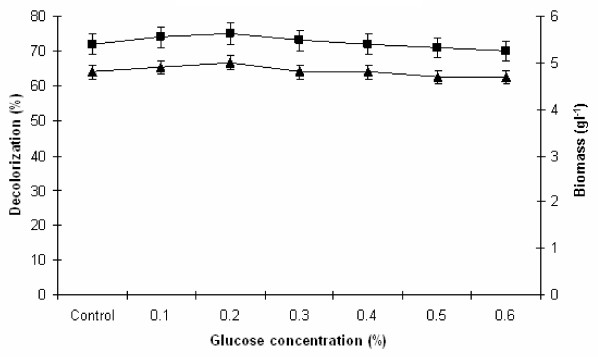
**Effect of different nitrogen sources on melanoidin decolorization.** Decolorization yield (), Biomass production (). The control flask does not contain any nitrogen sources. Test flasks contained different nitrogen sources in the medium at a level of 0.5% (w/v). Inoculated flasks were incubated at 45°C for 24 h. Error bars presented are mean values of ± standard deviation of triplicates of three independent experiments.

The influence of various organic and inorganic nitrogen sources (0.5%, w/v) on melanoidin decolorizatin and biomass production was also investigated for 24 h of incubation, and the results are depicted in Figure 7. Melanoidin decolorization by yeast strain Y-9 is extraordinarily stable in the presence of all nitrogen sources under study. It was observed that except sodium nitrate and beef extract, presence of other nitrogen sources enhanced the melanoidin decolorization when compared to control (without nitrogen source). From Figure 7 it observed that yeast strain showed higher 75% decolorization with 4.95 g l^−1^ biomass production in the presence of peptone, it was practically high compared to the extent of decolorization reported by other workers [8]. Sirianuntapiboon et al. [28] have reported that yeast extract and peptone inducing decolorizing activity in acetogenic bacterium strain No. BP103. But in case of *Issatchenkia orientalis* and *Citeromyces* sp. WR-43-6, maximum decolorization was reported in the presence of 0.1% NH_4_Cl and 0.1% sodium nitrate [19,20]. Kirk et al. [29] have reported that enzymatic systems catalyze degradation of lignin and lignin-like materials during the secondary phase of the metabolic growth in the presence of peptone. Synthesis and secretion of lignin peroxidase or ligninase (LiP) and manganese-dependent peroxidase (MnP) are triggered by nutrient limitations such as carbon and nitrogen sources.

### Effect of different concentration of peptone on melanoidin decolorization

**Figure 8:**
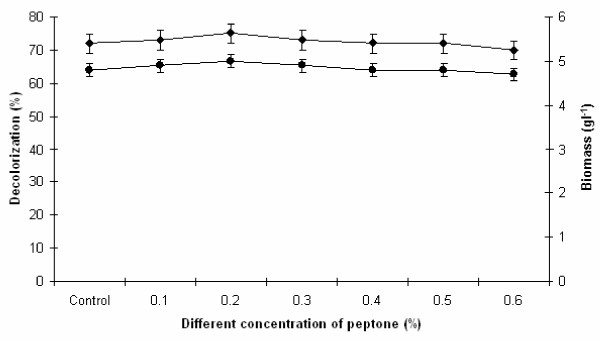
**Effect of different peptone concentration on melanoidin decolorization.** Decolorization yield (), Biomass production (). The control flask does not contain peptone. Test flasks contained different concentration of peptone in the medium at a level of 0.6% (w/v). Inoculated flasks were incubated at 45°C for 24 h. Error bars presented are mean values of ± standard deviation of triplicates of three independent experiments.

In another set of the experiment, the effect of various concentration of peptone (0.1, 0.2, 0.3, 0.4, 0.5 and 0.6%, w/v) on melanoidin decolorization and biomass production was also investigated and the results are depicted in Figure 8. Melanoidin decolorization by yeast strain Y-9 is extremely stable in the presence of all peptone concentrations under study. It was observed that peptone concentration above 0.3% (w/v), decreased the melanoidin decolorization. From Figure 8 it was observed that maximum 75% decolorization with 5.0 g l^−1^ biomass production was achieved at 0.2% (w/v) peptone concentration, above this concentration reduced decolorization. Similarly Ravikumar et al. [26] have also reported that *cladosporium cladosporioides* showed maximum decolorization at low concentration of peptone (1.0 g l^−1^) because at high concentration there was no significance in decolorization due to surplus supplementation of nitrogen which inhibition the growth. Similar effect was observed when low concentration of peptone was used as nitrogen source by *Phanerochaete Chrysosporium* for decolorization of melanoidin pigment present in spentwash [5]. It is, therefore, evident from our study that melanoidin decolorization by yeast strain Y-9 is remarkably higher in the presence of 0.2% (w/v) peptone within 24 h of incubation. This culture utilized little amount of peptone for higher melanoidin decolorization compared to other researchers ever reported.

### HPLC analysis

**Figure 9:**
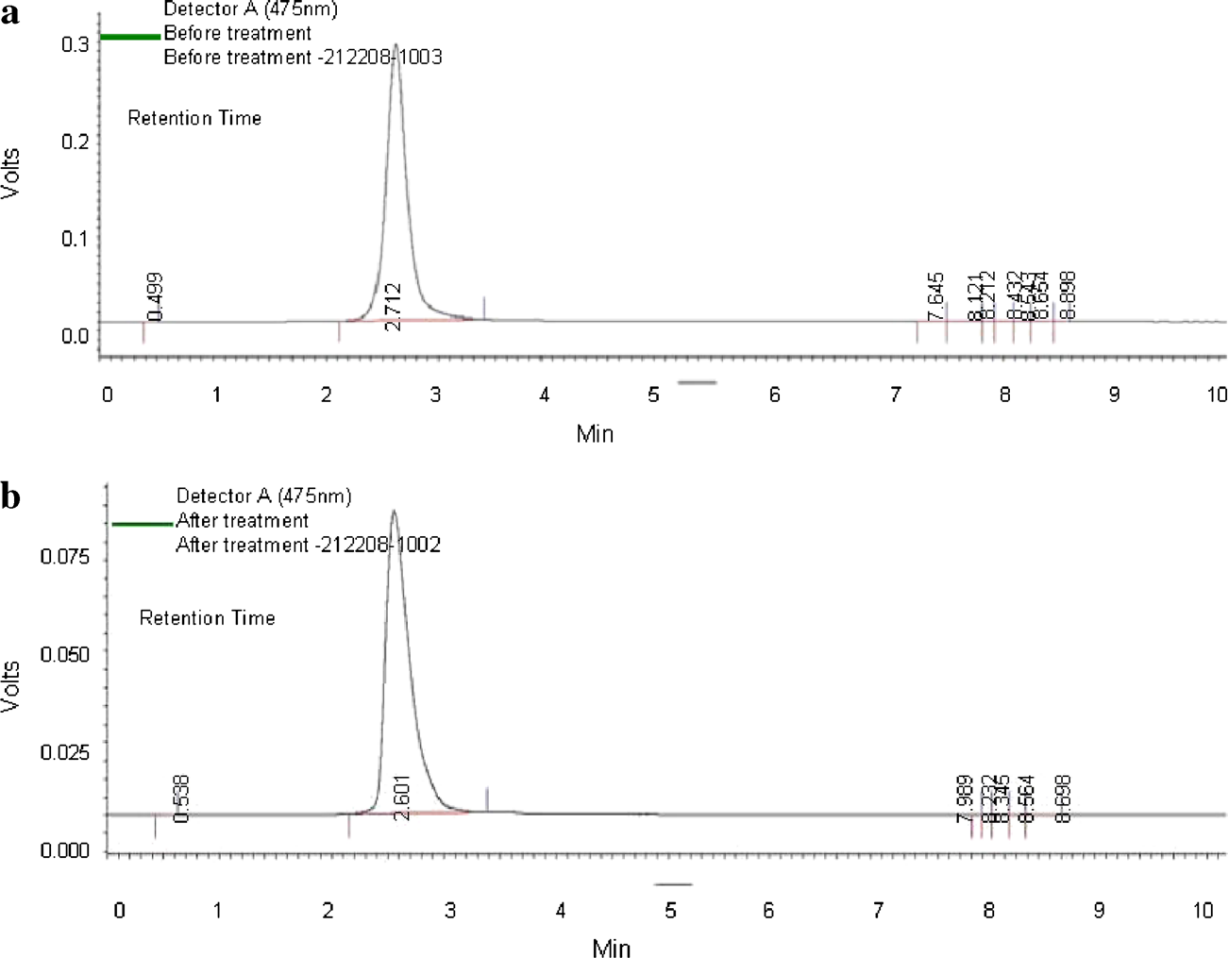
**HPLC analysis for monitoring the spentwash decolorization. 9(A)** HPLC analysis for distillery spentwash before treatment showing a maximum peak with 2.712 retention time, 3916140 area and 276697 height. **9(B)** HPLC analysis for distillery spentwash after treatment showing a maximum peak with 2.601 retention time, 1253879 area and 84452 height.

The HPLC analysis report representing the area, height, retention time, before and after the treatment of spentwash (Figure 9a and b) which confirms the biodegradation of melanoidin, main compound/pigment responsible for dark brown color of distillery effluent. A major peak appeared at a retention time of 2.60 min in treated sample which was less compared to untreated and clearly indicates the ability of the yeast to decolorize/degrade the spentwash. The reduction in physico-chemical characteristics may be due to degradation of melanoidin in the presence of carbon and nitrogen sources through metabolism.

## Conclusion

The thermotolerant *Candida tropicalis* has ability to decolorized complex melanoidin compound at wide range of temperature and pH in presence of little amount of carbon and nitrogen sources within a short incubation period of 24 h. This strain has ability to reduce environment pollution by decolorizing melanoidin pigment with cost effective and eco-friendly nature.

## Materials and methods

### Distillery spent wash (DSW)

The molasses spent wash was collected aseptically from Masuadha sugarcane distillery, faizabad U.P., India. The spentwash was centrifuged at 10,000 rpm for 15 min before use to remove the suspended solids and stored at 4°C. The stored distillery spentwash was filtered through (Whatman No: 1) filter paper and was diluted with distilled water [25]. The analysis of different physico-chemical parameters like color, odor, pH, biological oxygen demand (BOD), chemical oxygen demand (COD), total sugars, total dissolved solids (TDS), sulphates, phosphorous and calcium were analyzed for employing standard methods for examination of water and wastewater [30] and is shown in Table 1.

**Table 1:** Physico-chemical properties of distillery effluent (spentwash)

**Parameters**	**Value of distillery effluent**
Color	Dark brown
Odour	Like molasses
Temperature °C	82
pH	4.2
Total dissolved solid (mg l^−1^)	81733
Total suspended solid (mg l^−1^)	5933
Dissolved oxygen (mg l^−1^)	0
Biological oxygen demand (mg l^−1^)	46666
Chemical oxygen demand (mg l^−1^)	104130
Total nitrogen (mg l^−1^)	1635
Phosphorus (mg l^−1^)	163
Potassium (mg l^−1^)	8766
Sodium (mg l^−1^)	211
Calcium (mg l^−1^)	1816
Sulphate (mg l^−1^)	1738

### Isolation, screening and identification of melanoidin-decolorizing yeast

Melanoidin decolorizing yeast isolated from soil sample collected from distillery was grown on GPYE agar medium for 24 to 48 h incubation. Culture medium consisted of 0.2%, K_2_HPO_4_; 0.1%, KH_2_PO_4_; 0.01%, MgSO_4_.12H_2_O; 0.5%, glucose and 0.1%, yeast extract with 3.5 OD effluent and the initial pH was adjusted to 5.5. In order to isolate molasses decolorizing yeast, 1.0 g of soil was serially dilution upto 10^−5^ to 10^−6^ and placed in Petri plates along with the GPYE agar medium. The plates were subsequently incubated for 24–48 h at 35 ± 2°C and 45 ± 2°C for thermotolerant yeast. After 24–48 h of incubation, decolorization efficiency was recorded visually. The isolates showing more decolorization of the melanoidin were selected for further studies, maintained on the same medium at 4°C in slants, and sub-cultured after two weeks. These cultures were identified at genus and species level by Institute of Microbial Technology (IMTECH) MTCC Chandigarh, India.

### Inoculum preparation

Mother culture was prepared by inoculating one full loop of 24 h grown culture on basal agar plate in 50 ml basal broth, and incubated at 37°C for 24 h to achieve active exponential phase consisting of 50x10^6^ cfu ml^−1^ population. Appropriate volume (0.5%, v/v) of this cell suspension was used to inoculate the test flasks.

### Decolorization assay

The melanoidin decolorizing yeast was inoculated in the GPYE broth medium and after incubation; broth was centrifuged at 10,000 rpm for 10 min. The supernatant of the centrifuged sample was read at absorbance maximum (A_max_) of the melanoidin i.e. 475 nm using spectrophotometer [31]. The decolorization yield was expressed as the decrease in the absorbance at 475 nm against initial absorbance at the same wavelength. Uninoculated medium served as control. The entire assays were performed in triplicate and compared with control. The decolorization efficiency of the isolate was expressed as per following equation:

(1) $$\text{Decolorization}\;\left(\%\right)=\text{I}-\text{F}/\text{I}$$

Where, I = Initial absorbance (Control) and F = Absorbance of decolorized medium broth.

### Biomass determination

Yeast cells in broth were collected by centrifugation (10,000 rpm for 10 min at 4°C), washed with distilled water, and dried in an oven at 80°C until getting a constant dried weight reported in the form of dry cell mass (g l^−1^).

### Selection of efficient medium for melanoidin decolorization

An experiment was conducted to select a suitable medium for efficient decolorization by the yeast strain. The medium having various combinations of glucose, peptone and effluent was used to evaluate decolorization potential of the isolate. Three types of media with different composition were used to evaluate.

Medium A: - Distillery effluent without carbon and nitrogen supplemented medium with 3.5 OD.

Medium B:- 0.5%, glucose; 0.2%, yeast extract; 0.3%, peptone; 0.05%, MgSO_4_; 0.05%, K_2_HPO_4_ with 3.5 OD effluent.

Medium C: - 0.6%, glucose; 0.5%, peptone; 0.05%, MnSO_4_; 0.05%, K_2_HPO_4_ with 3.5 OD effluent respectively.

### Selection of Physico-chemical and nutritional parameters for melanoidin decolorization

#### Optimization of experimental conditions

The various process parameters influencing melanoidin decolorization and biomass production by fermentation were optimized individually and independently of the others, therefore, the optimized conditions were subsequently used in all the experiments in sequential order. For optimization, the basal medium contained glucose 0.5%; peptone 0.2%; yeast extract 0.3%; K_2_HPO_4_ 0.05% and MgSO4 0.05% with 3.5 OD spentwash at pH −5.5 was used for inoculation with 0.5% (v/v) of yeast culture having 50x10^6^ cfu ml-1 and then incubated for different periods viz. 8, 16, 24, 32, 40 and 48 h at different temperature viz. 25, 30, 35, 40, 45 and 50°C. For melanoidin decolorization all the experiments were carried out under static. Initial pH also plays an important role in melanoidin decolorization and biomass production, so pH of medium was adjusted to 4.0, 4.5, 5.0, 5.5, 6.0, 6.5 and 7.0 using either 1 N HCl or 1 N NaOH. For the optimal melanoidin decolorization and biomass production, the strains may require additional carbon and nitrogen sources with varying concentrations in its growth media. Therefore, the growth medium was supplemented with the carbon sources viz. glucose, fructose, sucrose, maltose, lactose and starch (at the level of 0.5%, w/v) and nitrogen sources viz. ammonium sulphate, yeast extract, peptone, beef extract, malt extract, sodium nitrate, and sodium nitrite (at the level of 0.5%, w/v). Thereafter, optimized carbon and nitrogen sources were further optimized at different concentration (0.1 to 0.6%, w/v). The fermentation medium was sterilized at 121°C for 15 min and incubation was done at 45°C with all the other conditions at the optimal levels determined previously.

### HPLC analysis of spentwash

Decolorization of melanoidin (spentwash) was monitored by HPLC (Shimadzu). 10 ml of samples were taken, and centrifuged, filtered through 0.45 μm membrane filter (Millipore). Filtered sample was analyzed using mobile phase consisting acetonitryl and methanol (45:55) (HPLC grade) with 1 ml glacial acid and 0.5 ml sodium acetate [26,32]. The sample was eluted using C-18; reverse phase column of 5 μm SGE, 250 x 4.6 mm SS. Resultant peak was analyzed with UV–detector 475 nm. The flow rate of the mobile phase was 1 ml min^−1^.

### Statistical analysis

All experiments were carried out in triplicates and the results are presented as the mean of three independent observations. Standard deviation for each experimental result was calculated using Microsoft Excel.

## Competing interests

The author(s) declare that they have no competing interests.

## Authors’ contributions

ST carried out the research work and drafted the manuscript. RS was involved in revising the manuscript critically for important intellectual content. RG has designed the experiment, contributed substantially to analysis and interpretation of data and have given final approval of the version to be published. All authors read and approved the final manuscript.
